# Transformation of Hand-Shape Features for a Biometric Identification Approach

**DOI:** 10.3390/s120100987

**Published:** 2012-01-16

**Authors:** Carlos M. Travieso, Juan Carlos Briceño, Jesús B. Alonso

**Affiliations:** 1 Signals and Communication Department, Institute for Technological Development and Innovation in Communications, University of Las Palmas de Gran Canaria, Campus Universitario de Tafira, Ed. de Telecomunicación, Pabellón B, Despacho 111., Las Palmas de Gran Canaria 35017, Spain; E-Mail: jalonso@dsc.ulpgc.es; 2 Computer Science Department, University of Costa Rica, San Jose, Costa Rica; E-Mail: juan.briceno@ucr.ac.cr

**Keywords:** hand-based biometrics, hand identification, DHMM kernel, supervised classification, image sensor, edge detection, biometrics

## Abstract

The present work presents a biometric identification system for hand shape identification. The different contours have been coded based on angular descriptions forming a Markov chain descriptor. Discrete Hidden Markov Models (DHMM), each representing a target identification class, have been trained with such chains. Features have been calculated from a kernel based on the HMM parameter descriptors. Finally, supervised Support Vector Machines were used to classify parameters from the DHMM kernel. First, the system was modelled using 60 users to tune the DHMM and DHMM_kernel+SVM configuration parameters and finally, the system was checked with the whole database (GPDS database, 144 users with 10 samples per class). Our experiments have obtained similar results in both cases, demonstrating a scalable, stable and robust system. Our experiments have achieved an upper success rate of 99.87% for the GPDS database using three hand samples per class in training mode, and seven hand samples in test mode. Secondly, the authors have verified their algorithms using another independent and public database (the UST database). Our approach has reached 100% and 99.92% success for right and left hand, respectively; showing the robustness and independence of our algorithms. This success was found using as features the transformation of 100 points hand shape with our DHMM kernel, and as classifier Support Vector Machines with linear separating functions, with similar success.

## 1. Introduction

Biometric systems are reaching more importance for human identification or as verification systems. This importance is manifest in both areas (research and business), due to their inherent advantages over carrying magnetic cards, for example, or passport or PIN number reminders, *etc*. Those elements can be forgotten, and moreover may be used by non-authorized persons. The use of identification based on human body is well accepted and perceived naturally by male and female persons. Therefore, biometric identification is achieving outstanding acceptance and helping advance the field. In biometrics, hand identification systems have attained great importance due to their simplicity and discrimination capacity [1]. Besides, the use of the hand requires a medium-low precision data representation and it has a high social acceptance. In the hand identification research area, the most used and studied systems are based on hand geometry features, and the building of statistic models [2–4]. In order to classify those parameters with robust systems, among others, neural networks and linear classifiers have been used. In another reference [5], the authors discuss the application of a fuzzy pattern recognition algorithm based on Lattice Similarity Degree in a Hand-shape Identity Recognition System. The success rate was 96.5%.

A hand geometry recognition method based on the Gaussian mixture model (GMM) used one-dimensional centroid distance series to describe two-dimensional hand geometry [6]. Each finger was separated and formed one centroid distance series respectively and then a Gaussian mixture model (GMM) for the centroid distance series of each finger was built to carry out hand shape classification and certification. The hand-shape images database used was collected by their laboratory, and consisted of fifty persons’ images and five images per person. The success rate achieved for hand shape recognition was 99.8%. In [7], the proposed scheme uses the Radon transform for extracting hand shape feature. The Radon transform is simply the line integral of an object on the image plane along all the lines from 0 to 360 degrees. The distance between center of mass of the hand palm and the boundary points on the hand is maximum at the middle finger tip. This fact is used to find the optimal parameter for Radon transform and one dimensional position invariant features are extracted from the binary hand silhouettes. The proposed scheme was tested on a data set of 136 images with simple Euclidian norm based match scoring. The method attained an Equal Error Rate (EER) of 5.1%.

Other authors have employed a global hand shape-based approach for person identification and verification using two methods [8]. The the first, the features consist of hand contour data, and a classifier based on modified Hausdorff distance was used; the success rate achieved in this case was 98.75%. In the second the features consist of independent components of the hand shape, and the Euclidean distance was used as classifier; success rates of up to 99.48% were achieved. The database was composed by 1,374 images extracted from 458 subjects (three images per subject). In [9], the authors proposed a method for hand shape verification based on HMM. They separate the whole hand into five fingers and model the HMM, transforming the whole hand verification into the verification of each finger, and each finger’s verification result is summarized. Each point of the contour is characterized by two parameters: (1) radius-contour point. (2) the curvature at the contour point. They used continuous and discrete HMM. A data set of 300 images of twenty six persons was collected. Each person had between nine and fifteen images. The success rate achieved was 90%.

Hands have many intra-modalities: palmprint, veins, knuckles, *etc*. [10,11], and sometimes, they are combined or fused, using hand-shape features, as one of the intra-modalities. An example is shown in [10]. This work proposes a new bimodal biometric system using feature-level fusion of hand shape and palm texture. The comparison and combination of proposed features is evaluated based on diverse classification schemes such as naive Bayes (normal, estimated, multinomial), decision trees, k-NN, SVM, and FFN. The feature selection strategy has been able to find 20(10) best features that give 96% (89%) accuracy using the SVM classifier. This 20(10) feature subset consists of 15(6) palmprint and 5(4) hand-shape features. The image database was collected from 100 subjects. The dataset consisted of 1,000 images, ten images per subject, which were obtained with a digital camera using an unconstrained peg-free setup in an indoor environment.

Another use of these shape-hand features is for gesture identification, as shown in [12]. Two problems were covered. The first concerns persons’ identification based on the shape of the hand using invariant geometrical features. The identification is achieved by computing the distance between two feature vectors of two hand images. The second is the recognition of gestures and signs made by hands. The hand gesture database was composed of 300 samples. The proposed approach, which uses gesture blob from features, texture and moment invariants based on Radon transform, correctly detected 282 hand gestures. The correct detection rate was 94%.

**Figure 1.**
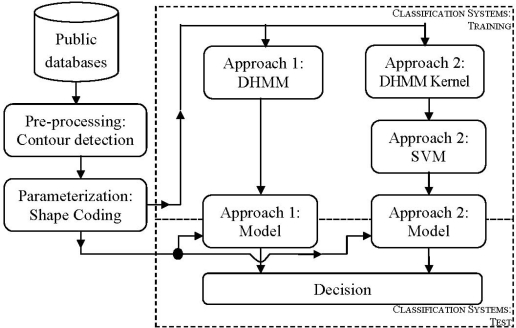
Flow chart of our proposal.

Contrary to those methods and using new parameterization techniques, our system has been designed in order to work only with edge features. In particular, in this work, the authors have implemented a system based on hand-shape features (see Figure 1). The approach has been developed as hand identification based on a DHMM kernel [13], and classified with Support Vector Machines (SVM) [14]. The advantages of SVM for this approach are due to its good behavior under short training data conditions, being our case when data are generated by the DHMM Kernel. The principal contribution of this work is the use of edge coding for hand-shape features, and its DHMMK transformation (see Figure 1). This proposal has been checked for two databases: our database and a public database, in order to check our algorithms. This is a good contribution to the state-of-the-art due to good results reached, and also, it contrasts with other methods used in the state-of-the-art.

The rest of this paper is organized as follows: Section 2 presents the databases, and Section 3 introduces the feature descriptors. Section 4 describes our different classification schemes. Experimental results are given in Section 5. In Section 6 a discussion is given. Conclusions about our work are presented in Section 7.

## 2. Databases

Our database (GPDS database) has been built with 144 users, acquiring 10 samples per class and has been built in three sessions. An HP scanner has been used in order to acquire each sample, hence minimizing the environment effects. Another public database has been used, the UST Hand Image database, which has been created by the Hong Kong University of Science and Technology [15]. This set contains 10 images of the right hand and 10 images of the left hand from 287 people. The hands have been acquired in a contactless scenario with an Olympus C-3020 digital camera (1,280 × 960 pixels) and they have not employed any special illumination or used any pegs. The following table shows the most important characteristics of both databases.

## 3. Shape Coding

**Figure 2.**
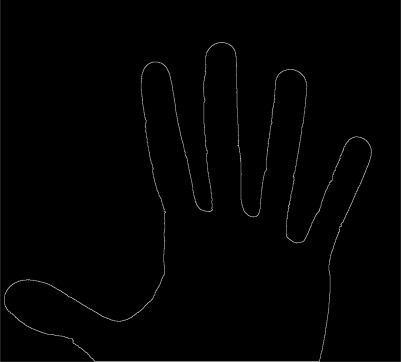
Example of a binarized hand shape image. The model is then used to perform (*x*,*y*) coding.

For the purposes of this study, only the hand shape has been considered. Images have been binarized by Otsu’s method [16] (Figure 2) and the contour is found by edge detection. This simple method is sufficient to detect the edges correctly.

Contour characterization by (*x*,*y*) positions of perimeter pixels, has been achieved firstly using a shadowing process (black shape over white background), then filtering isolated points, and finally, an automatic perimeter points location *x = line y = row* coding, with a subsequent point by point procedure. Finally, we obtain a perimeter description of {(*x_i_*, *y_i_*)| *i* = 1,…,*n*} points location description representing the closed border of a hand contour with one wide stroke pixel.

**Figure 3.**
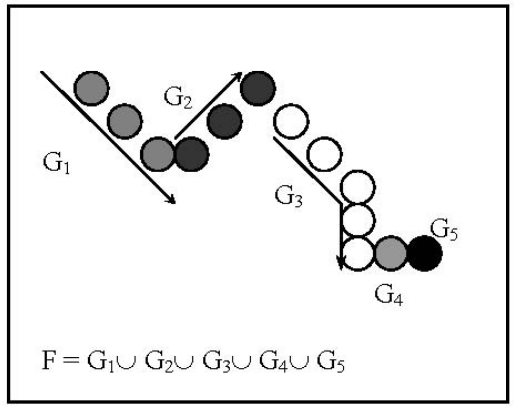
Example of a sequential trajectory decomposition. To avoid reduced trajectories to one point, *G*_4_ and *G*_5_ will be eliminated.

Data compression, size regularization and critical control point selection of perimeters description are achieved by a structuring procedure (Figure 3).

This procedure is based on the idea that a one pixel stroke on a black and white image may be described as a graph *G_f_* of a one dimensional trajectory application *f*, if preservation of a correct sequencing definition or monotonic behaviour on the *x* ordinate has been used. That is *G_f_* = {(*x_i_*, *y_j_*)| *y_i_* *= f*(*x_i_*), *i=* 1,…,*n*} where ordinate points *x_i_*, of the *f* stroke must be such that: *x_i_* *< x_i+1_* or *x_i+1_* *< x_i_* for *i* = 1,…,*n–*1. Afterwards, considering the complete perimeter *G,* its description relation *F* is defined as the partial definition of a piece like 1-D trajectory applications *f_j_* (with *G_j_* graphs) preserving monotonic behaviour. That is:

(1) $$G=\underset{j\subset J}{\cup }G_{j},$$

where *G_j_* is a set of positional border points, *G_j_* = {(*x_a_*, *y_a_*) | *y_a_* = *f_j_*(*x_a_*), *a* ∈ *J_j_*} for convenient sets of indexes *J* and *J_j_*. The restriction trajectory applications (or coded pieces of border) *f_j_* *= F*_{*x*|α∈*J*}_ are set in such a way that the next point following the last of *G_j_*, is the first of *G_j_*_+1_. Accordingly *G_j_* graphs are correct *f_j_* trajectory applications descriptions. To avoid *G_j_* being reduced to one pixel, only the first point of a constant *x* ordinate series is preserved. Note that the structure of the *G* border by partial graph descriptions *G_j_* accounts for abrupt direction changes in the perimeter description.

**Figure 4.**
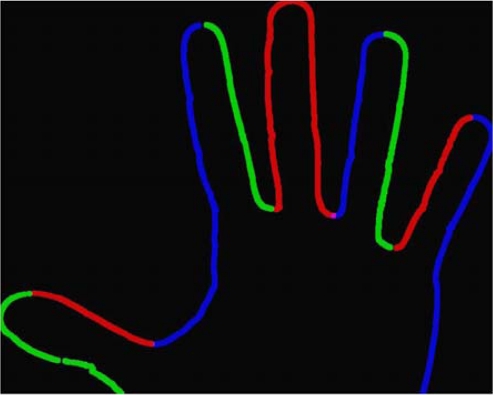
Example of a hand *G_j_* graph structure: starting the reading on the arrow, and then it follows counterclockwise (red, green, blue).

After building up the *G_j_* all the *n* first points of each *G_j_*, *j* = 1,…, *n* are selected and for an arbitrary constant number *p* ≥ *n* the perimeter points description is completed by *k = n–p* points, with uniform distribution for each *G_j_* and proportional to its size. An example of structured G*_j_* graph is presented in Figure 4.

In order to perform a rotational, scale size and origin reference-free coding an angle transformation is applied for the positional point border coded as before. For a given coded border of *n* positional control points *G* = {*X_i_* = (*x_i_,y_i_*)|*i* = 1,…, *n*} let *C_0_* be its central point, and let *β_i_* and α*_i_* be the angles; *β_i_* = angle(*C*_0_, *X_i_*, *X_i_*_+1_) and *α_i_* = angle(*X_i_*, *C*_0_, *X_i_*_+1_). Then the sequence of (*x_i_, y_i_*) *i =* 1,…,*n* positional points are transformed in a sequence of (*α_i_,β_i_*) *i* = 1,…, *n–*1 angular origin free representations points.

We note that the choice of the start point *X*_1_ and the *C*_0_ points account for scale and hand shape rotation. We note also that geometrical properties of triangular similarities make such hand shape coding sequence size and location free. Finally, we also note that points are locally dependent: two consecutive points are geometrically related one point to the other, and if this sequence is pattern related, statistically they will verify Markov dependencies and hence could be correctly Hidden Markov valuated.

## 4. HMM Kernel: Classification System

In order to implement the classification system based on a DHMM kernel from edge data, three steps must be followed: the first one is the use of Discrete Hidden Markov Model (DHMM) obtained from the hand contour, this idea has been used in [9], but in this work we have changed the shape coding. The second one is the transformation of the data with the HMM kernel, and finally the use of a Support Vector Machine (SVM) as classifier.

### 4.1. HMM

The HMM is the representation of a system in which, for each value that takes a variable *t*, called time, in one and only one of N possible states is found and this declares a certain value at the output. Furthermore, an HMM has two associated stochastic processes: one hidden one associated with the probability of transition between states (non-observable directly); and another observable one, associated with the probability of obtaining each of the possible values at the output, and this depends on the state in which the system has been found [17]. A Discrete HMM (DHMM) has been used, which is defined in [17,18].

N is the number of states, M is the number of different observations, *A*(*N*,*N*) is the transition probabilities matrix from one state to another, *π*(*N*,*1*) is the vector of probabilities that the system begins in one state or another; and *B*(*N*,*M*) is the probabilities matrix for each of the possible states of each of the possible observations being produced.

We have employed a DHMM called “left to right” or Bakis, which is particularly appropriate for sequence evaluation. These “left to right” DHMM’s turn out to be especially appropriate for hand shape recognition because the transition through the states is produced in a single direction, and therefore, it always advances during the transition of its states, which provides this type of model with the ability to maintain a certain order with respect to the observations produced where the temporary distance among the most representative ones changes. Finally, from 20 to 140 states and 32 symbols per state have been used.

In the DHMM approach, the conventional technique for quantifying features is applied. For each input vector, the quantifier decides which is the most convenient value from the information of the previous input vector. To avoid taking a software decision, a fixed decision on the value quantified is made. In order to expand the possible values that the quantifier is going to acquire, multi-labelling is used, so that the possible quantified values are controlled by varying this parameter. The number of labels in the DHMM is related to values that can be taken from the number of symbols per state.

DHMM algorithms should be generalized to be adjusted to the output multi-labeling ({*v_k_*} *k =* 1,…,C), to generate the output vector ({*w(x_t_,v_k_*)}*k =* 1,…,C). Therefore, for a given state *j* of DHMM, the probability that a vector *x_t_* is observed in the instant *t*, can be written as:

(2) $$b_{j}\;(x_{t})=\sum_{k-1}^{c}w(x_{t},\;v_{k})b_{j}\;(k)$$

where *b_j_*(*k*) is the output discrete probability, associated with the value *v_k_* and the state *j* and C is the size of the vector values codebook.

This approach has to model a DHMM from the hand contour; after experimentation, it can be observed that this system is not appropriate for achieving a discriminative identification system. Therefore, an improvement by the transformation of the DHMM kernel is proposed.

### 4.2. Data Transformation

The next step is the transformation of DHMM probabilities, related to the approach of the Kernel building [13]. With this goal, the aim is to unite the probability given by the DHMM to the given discrimination provided by the classifier based on SVM. This score calculates the gradient with respect to DHMM parameters, in particular, on the probabilities of emission of a vector of data *x*, while it is found in a certain state *q ∈* {*1*,...,*N*}, given by the matrix of symbol probability in state *q* [*b_q_*(*x*)], as indicated in Equation (2):

(3) $$P(x|q,\;\lambda )=b_{q}(x)$$

If the derivative of the logarithm of the previous probability is calculated (gradient calculation), the DHMM kernel (DHMMK) is obtained, whose expression is given by:

(4) $$\frac{\partial }{\partial P(x,\;q)}\text{log}\;P(x/q,\;\lambda )=\frac{\xi (x,\;q)}{b_{q}\;(x)}-\xi (q)$$

Approximations and calculations for the previous equation can been found in [13]. In our case, and using DHMM, *ξ*(*x,q*) represents the number of times that it is localized in a state *q*, during the generation of a sequence, emitting a certain symbol *x* [13,17]. *ξ*(*q*) represents the number of times which it has been in *q* during the process of sequence generation [13,17]. These values are directly obtained from the forward backward algorithm, applied to DHMM by [17,18].

The application of this score (*U_X_*) to the SVM is given by the expression of Equation (3), using the technique of the natural gradient (see Equation (4));

(5) $$U_{x}=\nabla_{P(x,q)}\text{log}\;P(x|q,\;\lambda )$$

where *U_X_* defines the direction of maximum slope, obtained from the logarithm of the probability of having a certain symbol in a state.

### 4.3. SVM Classification System

The goal consists of training the system to obtain two sets of vectors (in two dimensions corresponding with points) that represent the classes to identify. Subsequently, the separating hyperplane *H* (in two dimensions is a linear classifier) between these two sets is calculated. The pertinent points within the hyperplane have to satisfy the following Equation [19]:

(5) w×x+b=0

where *w* is normal to the hyperplane, *b/||w||* is the perpendicular distance from the hyperplane to the origin, ||*w*|| is the Euclidean norma, *b* is the independent term, and *x* is a point in the hyperplane. Furthermore, another two hyperplanes are defined as follows; *H*_1_*: x_i_·w + b =* 1 and *H*_2_*: x_i_w + b =* −1, which contains support vectors. The distance between planes *H*_1_ and *H*_2_ is known as the margin. The aim of this classification algorithm is to calculate the maximum of the mentioned margin.

Once the system has been trained and, therefore, the separation hyperplane has been obtained, we have to determine what the decision limit is (hyperplane *H* located between *H*_1_ and *H*_2_ and equidistant to them). In accordance with the previous decision, the corresponding class label is assigned, that is, the class of *x* will be defined by *sgn*(*w·x+b*). This means that test samples are assigned with label “+1”, and the remainder, with label “−1”.

SVM calculates the separation between classes, by means of the calculation of the natural distance between the scores of two sequences *X* and *Y*:

(7) $$D^{2}(X,\;Y)=\frac{1}{2}{\left(U_{X}-U_{Y}\right)}^{T}F^{-1}(U_{X}-U_{Y})$$

where *F* is the DHMM information matrix, and is equivalent to the matrix of covariance of the vectors *U_X_* and *U_Y_*.

Finally, different types of functions, which can be used for SVM, are with a linear and Gaussian kernel (RBF). This is used for establishing the decision limit. The RBF kernel is shown in the following equation:

(8) $$K(X,\;Y)=e^{-D^{2}(X,\;Y)}$$

Support Vector Machines (SVM) are a bi-class system, in other words only two classes are considered. In particular for this present work, this has been done with 144 classes, and for this reason, we have built a multi-class SVM with the one-versus-all strategy, like in [19]. This strategy is built from a bi-class classifier, the class under identification *vs*. the rest of the classes. It is done for all classes and finally the max score value will select the class to choose.

## 5. Experiments and Results

All experiments have been five-fold cross validated, and successes are shown for our tables of success rate, an mean and standard deviation based on identification, using a supervised classification. For a first round of testing, only 60 classes of GPDS database have been employed, from four to one hand samples for use in training mode, and then performance is tested with the rest (from six to nine). The idea is to observe the behavior of our approach using a short database. After obtaining the parameter tune ups, a second round test has been implemented for the both different classifiers (DHMM and SVM), with the entire collection of 144 classes in order to prove scalability and stability of the method. Finally, the third round of testing will check our approach with another public and independent database, in order to observe the robustness of our proposal.

Experiments have been based on two approaches. The first one, based on hand contour, was classified with the DHMM. The second one, built with an approach parameterized with the DHMM kernel, was classified with the SVM. Therefore, our results were obtained varying some parameters from the proposed systems; in particular, the number of HMM states (between 20 and 140 states) and two different kernels of the SVM, in particular, linear and Gaussian kernels.

For our first approach, in the first testing round the success rates achieved were less than 85% for DHMM states between 20 and 140. Table 2 shows the success classified with the DHMM from hand contour. Only the best results are shown with values from the DHMM (mean ± standard deviation). In the second approach, the previous results have been improved, introducing the DHMM score and the SVM. Table 3 shows the success classified with the SVM from the DHMM kernel (mean ± standard deviation), where gamma is the value to adjust the RBF separating functions.

For the second testing round (144 users—GPDS database), the previous results obtained with the HMM parameters have mainly been used: number of states, and number of contour sequences point descriptors; and for the SVM, the gamma adjusting RBF functions parameter. This allows us observe the robustness of our approach when the number of users is increased. Results are shown in Tables 4 and 5 only for five training samples. It is observed that the response of our approach, when the number of users is increased, keeps the same adjustment, done for 60 users, only a little change is detected for gamma. It shows that a minimum adjustment, our proposal can be working under good conditions.

Table 6 shows the success rates when the number of training samples is decreased (from five to one training sample) for 144 users, considering our best model; 100 edges coding points and transformed by 60 HMM states.

Comparing Tables 2 and 4, it is observed that the success rates are less for 144 users, but with only a small decrease (0.09%) when the number of users is increased by 130%. Therefore, this proposal shows and maintains robustness when the number of users is increased. Finally, experiments using the DHMMK+SVM classifier have also been performed to ascertain the performance for user authentication.

For the third testing round, the UST Database, with 287 users for left and right hands has been used. Our approach based on DHMMK+SVM has been checked, training the model only from 4 to 1 samples. The success rates are shown in Table 7.

**Figure 5.**
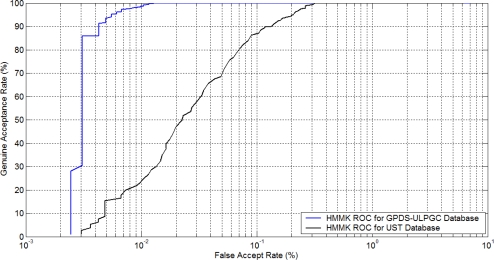
ROC curve for GPDS and UST databases under our best model based on DHMMK using two training samples (better viewed in color).

Besides, a similar biometric experiment protocol has been followed in [10] and the experiments for the above three different approaches performed to ascertain the performance of the proposed approach for user authentication. The authentication experiments are carried out on the two datasets respectively. Thus in total, two receiver operating characteristics (ROC) curves have been produced. Figure 5 shows the receiver operating characteristics curves of the approaches using DHMMK-SVM 60 DHMM states and 100 shape coding points using two training images per class on the GPDS and UST datasets.

Low resolution of the extracted hand contours tends to degrade the performance of different approaches. However, the level of degradation is different for each database; the number of users is another variable which affects this degradation. For the proposed approach using DHMMK, the performance is only slightly lowered, from 99.71% to 99.72%. This performance trend can now be seen from the ROC curves in Figure 5. This confirms that the proposed approach tends to be much more robust for resolution changes. Furthermore, a comparison *versus* references of the state-of-the-art is shown, in order to see the robustness of this work (see Table 8).

## 6. Discussion

After experiments, it has been considered that raw hand edge information is not a good classifying feature. Nowadays, many scientific references use other features as hand geometry (width of fingers, distances, *etc.*), palmprints, texture of fingers, knuckles, veins, *etc.*, but there are only a few papers about shape, because it is very difficult to obtain good results, and hence success rates are low *versus* other references. Therefore, contour transformation using the DHMM kernel has been introduced.

Each state in DHMM represents a contour variation, and the best discriminative system has 60 states from 100 points of contour description for our tuning procedure. A set of one, two or three points represent a state, as average. As it has low success, then the DHMM kernel has been applied as an enlarged representation, using the relation between *b_q_*(*x*), *ξ*(*x,q*) and *ξ*(*q*), according to Equation (4) of the HMM kernel.

Therefore, now the number of times that it is localized in a state *q*, the data vector for each state according the probability of emission for the same data vector for each state is being represented, and it is an enlarged representation. These new features have a large dataset and it is classified by SVM, due to its good behavior with big sets of features [14,19].

Success rates are shown in Table 3, and it has been demonstrated that the DHMM kernel is a very good and robust parameterization system. It is also shown that working with 100 edge points and using 60 DHMM states, SVM classification has resulted in the best success rates with the DHMM kernel. RBF and linear kernels can be done; the success has been the same, therefore, it is better to use a linear kernel because it is faster. Finally, for these case (60 classes), our proposal has achieved successes over 99.96%, with two hands as training samples (see Table 3).

After tuning, with a substantial data set augmentation to 144 users (about 2.4), similar results have been obtained with the same parameters *vs*. the reduced data set (60 classes), as shown in Table 5. For 100 points of contour descriptors, 60 states of DHMM representation and gamma value (4 × 10^−4^) similar success rates, about 99.71%, with linear as well as with RBF functions (using two training samples). As expected the number of training samples has been decreased from five to one in order to maintain similar performance. With the implementation of the second round of tests and obtaining similar results with about the same system parameters applied to the augmented 2.4 data set, system scalability and very good stability and performance have been shown.

In our third round of tests our approach has displayed a similar behavior, and has maintained the success rates with a low resolution (see Table 1) and increasing the number of users up to 287. For UST database, up to 99.92% success rate has been reached training with three hands. The similar success of GPDS and UST databases proves the good behavior and stability of our proposal.

## 7. Conclusions

An original and robust approach has been built for automatic hand-shape recognition, using the transformation of hand edges using HMM kernel, and classification with an SVM. The success rates are over 99.87%, working with the GPDS database, and with only three hand training sample for 144 users; and 99.92% for the UST database. The use of independent and public database gives robustness to our approach. In future works, the authors plan to use hand intra-modality information and apply data and score fusion. Finally, our approach will be checked against other public databases.

## Figures and Tables

**Table 1.** Characteristics of both databases.

**Parameters**	**Details from GPDS Database**	**Details from UST Database**
Number of classes	144	287
Number of samples per classes	10 right hand samples	10 left and 10 right hand samples
Acquisition and Quantification	Gray Scale (8 bits, 256 levels)	Gray Scale (8 bits, 256 levels)
Resolution	150 dpi	500 dpi
Size	1,403 × 1,021 pixels	1,280 × 960 pixels
Example	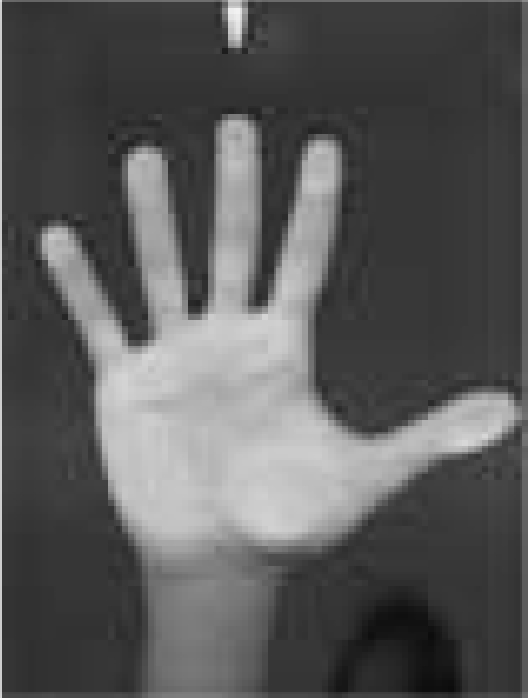	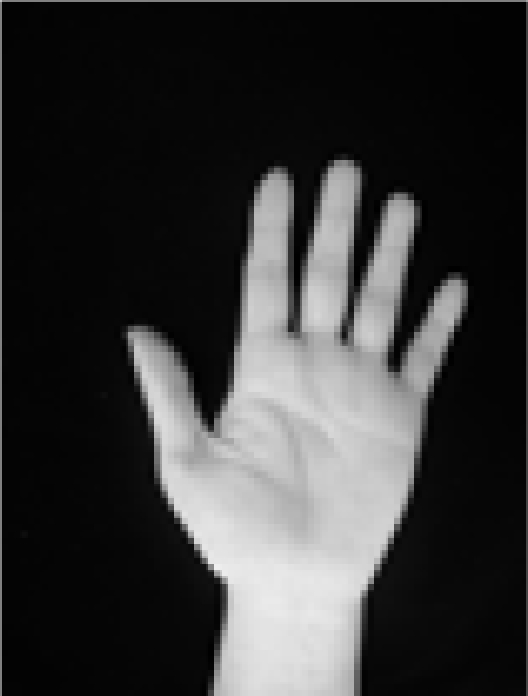

**Table 2.** Success rates for DHMM classifier, using edge information for 60 users.

**Number of Edge Points**	**DHMM States**	**Number of Samples Training**	**Success Rates**
750	60	4	46.61% ± 5.94
		3	38.09% ± 4.73
		2	32.41% ± 4.67
		1	23.55% ± 3.73

300	60	4	72.50% ± 4.77
		3	71.90% ± 4.36
		2	61.04% ± 6.48
		1	54.37% ± 5.71

200	60	4	84.17% ± 6.40
		3	82.33% ± 4.33
		2	79.79% ± 4.03
		1	73.92% ± 5.64

100	60	4	73.12% ± 6.21
		3	71.00% ± 7.84
		2	70.33% ± 2.33
		1	65.63% ± 5.63

**Table 3.** Success rates for the SVM classifier, using HMM kernel for 60 users.

**Number of Edge Points**	**Number of Samples Training**	**SVM (Success Rates)**		
		**Linear Kernel**	**RBF Kernel**	**Gamma**
750	4	100% ± 0	100% ± 0	1 × 10^−6^
	3	99.96% ± 0.08	99.96% ± 0.08	1 × 10^−6^
	2	99.96% ± 0.09	99.96% ± 0.09	1 × 10^−5^
	1	99.85% ± 0.16	99.85% ± 0.16	1 × 10^−6^

300	4	100% ± 0	100% ± 0	1 × 10^−6^
	3	99.93% ± 0.15	99.93% ± 0.15	1 × 10^−6^
	2	99.96% ± 0.09	99.96% ± 0.09	1 × 10^−6^
	1	99.85% ± 0.16	99.85% ± 0.16	1 × 10^−6^

200	4	100% ± 0	100% ± 0	1 × 10^−6^
	3	99.95% ± 0.11	99.95% ± 0.11	5 × 10^−6^
	2	99.96% ± 0.09	99.96% ± 0.09	8 × 10^−8^
	1	99.92% ± 0.10	99.92% ± 0.10	4 × 10^−8^

100	4	99.96% ± 0.09	99.96% ± 0.09	1 × 10^−6^
	3	99.95% ± 0.11	99.95% ± 0.11	1 × 10^−6^
	2	99.96% ± 0.09	99.96% ± 0.09	1 × 10^−6^
	1	99.85% ± 0.16	99.85% ± 0.16	1 × 10^−6^

**Table 4.** Success rates for the DHMM with 144 users.

**Number of Points**	**Number of States**	**Success Rates DHMM**
100	40	61.87% ± 1.75
100	50	62.24% ± 1.47
100	60	62.37% ± 0.50
100	70	61.72% ± 2.85
200	40	62.10% ± 1.33
200	50	67.74% ± 4.75
200	60	76.81% ± 3.35
200	70	81.21% ± 4.46
300	40	36.17% ± 4.61
300	50	51.19% ± 5.54
300	60	64.29% ± 6.27
300	70	69.98% ± 5.08

**Table 5.** Success rates for DKMM transformation and SVM with 144 users.

**Number of points**	**Number of states**	**Linear SVM**	**RBF SVM**	**gamma**
100	50	99.86% ± 0.14	99.86% ± 0.14	4 × 10^−6^
100	60	99.95% ± 0.11	100% ± 0	4 × 10^−6^
100	70	99.91% ± 0.08	99.91% ± 0.08	4 × 10^−6^
200	50	99.77% ± 0.08	99.77% ± 0.08	4 × 10^−6^
200	60	99.95% ± 0.08	99.95% ± 0.08	4 × 10^−6^
200	70	99.77% ± 0.08	99.77% ± 0.08	4 × 10^−6^
300	50	99.81% ± 0.08	99.81% ± 0.08	6 × 10^−7^
300	60	99.86% ± 0.01	99.86% ± 0.01	6 × 10^−7^
300	70	99.91% ± 0.08	99.91% ± 0.08	6 × 10^−7^

**Table 6.** Success rates for SVM with 144 GPDS users for 60 DHMM states and 100 edges coding points, decreasing the training samples.

**Number of Points**	**Number of Samples Training**	**Linear SVM**	**RBF SVM**	**Gamma**
100	5	100% ± 0	100% ± 0	4 × 10^−6^
100	4	99.92% ± 0.07	99.92% ± 0.07	4 × 10^−6^
100	3	99.87% ± 0.12	99.87% ± 0.12	4 × 10^−6^
100	2	99.71% ± 0.10	99.71% ± 0.10	4 × 10^−6^
100	1	99.42% ± 0.21	99.42% ± 0.21	4 × 10^−6^

**Table 7.** Success rates for SVM with 287 UST users for 60 DHMM states and 100 edges coding points, decreasing the training samples.

**Number of Points**	**Number of Samples Training**	**Linear SVM**	**RBF SVM**	**Gamma**
100	4 (left hand)	100% ± 0	100% ± 0	4 × 10^−6^
100	4 (right hand)	100% ± 0	100% ± 0	4 × 10^−6^
100	3 (left hand)	99.92% ± 0.17	99.92% ± 0.17	4 × 10^−6^
100	3 (right hand)	100% ± 0	100% ± 0	4 × 10^−6^
100	2 (left hand)	99.57% ± 0.44	99.67% ± 0.14	4 × 10^−6^
100	2 (right hand)	99.72% ± 0.07	99.72% ± 0.07	4 × 10^−6^
100	1 (left hand)	99.30% ± 0.12	99.34% ± 0.13	6 × 10^−6^
100	1 (right hand)	99.47% ± 0.07	99.59% ± 0.17	4 × 10^−6^

**Table 8.** Comparison with the state-of-the-art, for references which use hand-shape features.

**Reference**	**Method**	**Database Size (users)**	**Success**
This work	DHMMK + SVM	287 (UST database)	100%
This work	DHMMK + SVM	144 (GPDS-ULPGC Database)	100%
[8]	modified Hausdorff distance	458	99.48%
[5]	Lattice Similarity Degree	100	96.5%
[10]	hand-shape features + Naïve Bayes	100	96%
[9]	Geometric features + HMM	26	90%
